# Analysis of Predictive Risk Factors in Aquaporin-4-IgG Positive Highly Active Neuromyelitis Optica Spectrum Disorders

**DOI:** 10.3389/fneur.2021.731835

**Published:** 2021-08-26

**Authors:** Yanfei Li, Jinwei Zhang, Yongyan Zhou, Haojie Xie, Ranran Duan, Lijun Jing, Yaobing Yao, Junfang Teng, Yanjie Jia

**Affiliations:** Department of Neurology, The First Affiliated Hospital of Zhengzhou University, Zhengzhou, China

**Keywords:** neuromyelitis optica spectrum disorders, aquaporin-4, relapse, risk factors, comorbidities, homocysteine levels, 24h IgG synthesis rates

## Abstract

Neuromyelitis optica spectrum disorders (NMOSDs) are inflammatory diseases with a high risk of recurrence and progressive disability, and it is crucial to find sensitive and reliable biomarkers for prognosis and the early prediction of relapse. Highly active NMOSD is defined as two or more clinical relapses within a 12-month period. In this study, we analyzed independent risk factors among patients with aquaporin-4 (AQP4)-IgG positive highly active NMOSD. In this retrospective study, we analyzed the data of 94 AQP4-IgG positive patients with highly active NMOSD and 105 AQP4-IgG positive controls with non-highly active NMOSD. In order to rule out possible effects of previous treatments (such as glucocorticoids, immunoglobulin, and immunosuppressants), we focused on the first-attack NMOSD patients admitted to our hospital. Clinical data, including the age of onset, gender, comorbidities, and serum analysis and cerebrospinal fluid (CSF) analysis results, were collected, after which logistic regression models were used to determine the associations between the clinical factors and relapse outcomes. The prevalence of connective tissue disease and the proportion of antinuclear antibody (ANA)-positivity were higher in the highly active NMOSD group than in the control group. The leukocyte counts, homocysteine (Hcy) levels, CSF leukocyte counts, protein concentrations, IgG indexes, and 24h IgG synthesis rates were also higher in the highly active NMOSD group. The results of multivariate analysis indicated that connective tissue disease comorbidity (OR = 5.953, 95% CI: 1.221–29.034, *P* = 0.027), Hcy levels (OR = 1.063, 95% CI: 1.003–1.126, *P* = 0.04), and 24h IgG synthesis rate (OR = 1.038, 95% CI: 1.003–1.075, *P* = 0.034) may be independent risk factors for AQP4-IgG positive highly active NMOSD relapse after adjusting for various variables. Comorbidity of connective tissue disease, Hcy levels, and 24h IgG synthesis rate may be independent risk factors for AQP4-IgG positive highly active NMOSD.

## Introduction

Neuromyelitis optica spectrum disorders (NMOSD) are autoimmune diseases characterized by unpredictable attacks on the optic nerves, spinal cord, brainstem, and other areas of the central nervous system, which result in an accumulation of neurological disability. NMOSD has a wide spectrum of clinical features, including optic neuritis, longitudinally extensive transverse myelitis, diencephalic syndrome, and other encephalitic presentations. Patients with NMOSD have a high risk of recurrence and a high incidence of disability. The prognosis of relapsing NMOSD is usually poor, particularly among patients with frequent relapses (1–3). Therefore, researchers have attempted to find reliable and sensitive markers to predict NMOSD relapses and prognosis.

Highly active NMOSD, also known as highly relapsing NMOSD, is defined as at least two clinical relapses during the previous 12 months. Previous studies showed that the comorbidity burden was significantly higher among patients with highly active NMOSD compared with the overall NMOSD population (4, 5). However, the association between comorbidity and relapse has not been further analyzed.

Several predictive factors for the prognosis of NMOSD have previously been identified. For example, antinuclear antibodies (ANAs) were found to be related to more severe disease activity in NMOSD patients (6). Disease duration of NMOSD was shorter in ANA (+) patients with an Expanded Disability Status Scale (EDSS) value <4 than in ANA (–) patients (7). Serum homocysteine (Hcy) levels were significantly higher in patients with NMOSD with an EDSS value ≥ 4 in the acute stage, indicating that Hcy may play an important role in the progression of NMOSD (8). However, no prior study has comprehensively investigated the risk factors affecting patients with highly active NMOSD in a real-world setting.

In this study, we conducted a retrospective analysis to explore predictive risk factors among patients with aquaporin-4 (AQP4)-IgG positive highly active NMOSD. We focused on the first-attack NMOSD patients admitted to our hospital in order to rule out possible effects of previous treatments (such as glucocorticoids, immunoglobulin, and immunosuppressants) and accurately calculate the times of relapse events during the follow-up.

## Methods

### Participants

**Figure 1:**
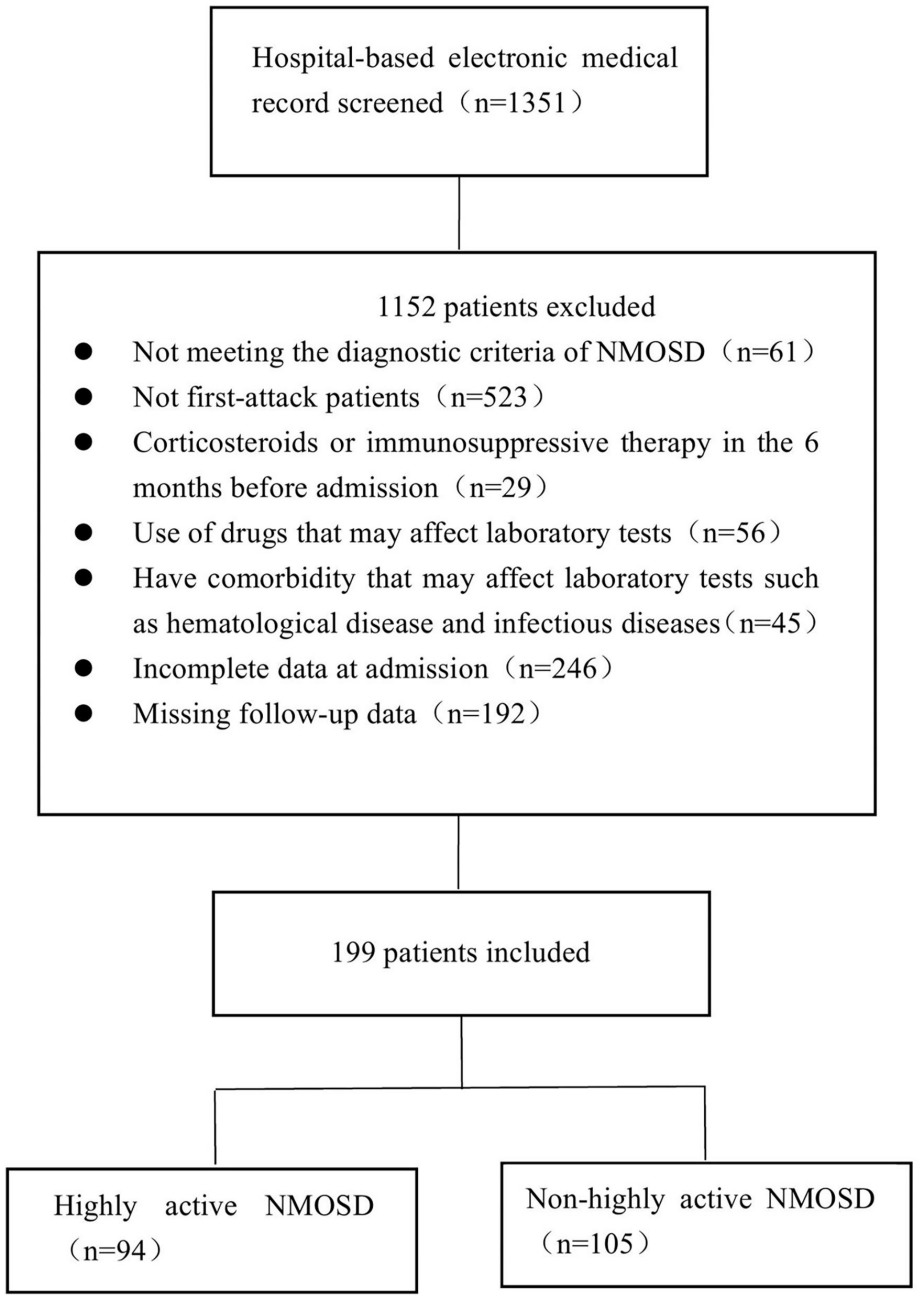
Overview of the patient selection process.

Patients diagnosed with NMOSD at the First Affiliated Hospital of Zhengzhou University between January 2013 and December 2019 were enrolled in this study. NMOSD was diagnosed based on the 2015 International Consensus Diagnostic Criteria for NMOSD (9). We screened patients who had experienced their first attack and divided them into two groups according to the number of relapses: patients who experienced two or more clinical relapses within 12 months following the first attack during the follow-up period were assigned to the highly active NMOSD group, while patients with no or one relapse within 12 months after the first attack were assigned to the control group (non-highly active NMOSD group). The exclusion criteria for both groups were as follows: (1) patients who had previously experienced NMOSD; (2) the coexistence of other diseases that may affect EDSS values; (3) taking corticosteroids or undergoing immunosuppressive therapy during the 6 months before admission; (4). using drugs that may affect laboratory tests such as lipid-lowering drugs, Hcy-lowering drugs, and hepatic and renal protectants before admission; (5) hematological, infectious, or other diseases that may affect blood test results and cerebrospinal fluid (CSF) analysis; (6) incomplete data at admission; (7) missing follow-up data; (8) patients who were AQP4-IgG negative or who were not tested. The detailed selection process is shown in Figure 1. The study was approved by the Ethics Committee of the First Affiliated Hospital of Zhengzhou University (2019-KY-018) and was performed in accordance with the Declaration of Helsinki.

### Data Collection

The patients' clinical data, including age of onset, gender, comorbidities, clinical symptoms, treatment, and laboratory test results (routine blood test, blood lipid, liver function, renal function, and thyroid hormone test, ANAs) and CSF analysis results (cells count, protein levels, IgG index and 24h synthesis rate) were collected. Blood samples were collected from patients after overnight fasting at 7:00–8:00 a.m. the next day after admission. Both blood and CSF samples were taken prior to treatment. Intrathecal immunoglobulin synthesis was detected using isoelectric focusing as previously described (10). The IgG index was calculated as the quotient of CSF-plasma concentration IgG divided by the CSF-plasma concentration quotient for albumin (QIgG/Qalb) (11). Anti-AQP4 antibodies in the serum or CSF were detected by an assay on live cells transfected with AQP4 at the Neurology Laboratory of the First Affiliated Hospital of Zhengzhou University (12). Other tests were performed in accordance with the manufacturer's protocols, and the examiners were blinded to the diagnoses and clinical symptoms.

Patients received different treatments according to their clinical symptoms and financial situation. Intravenous methylprednisolone or plasma exchange were used in most patients in the acute phase. Low dose oral prednisolone, azathioprine (2–3 mg/kg daily), mycophenolate mofetil (1–2 g daily), methotrexate (17.5 mg weekly) and rituximab were also used in part of patients to prevent attacks.

### Outcomes

The primary outcome of this study was highly active relapse events. A relapse event was defined as new-onset or recurrent neurological symptoms that lasted for at least 24 h and caused an EDSS increase of at least 0.5 points from the lowest score. Relapse events occurring within 28 days were regarded as part of a single relapse (13). Each patient was diagnosed and had their EDSS scores evaluated by an experienced neurologist. Clinical symptoms at the time of patients' first attack before treatment were recorded as initial EDSS scores. Clinical symptoms at the final follow-up were recorded as final EDSS scores. Follow-up data were obtained through an annual clinic visit or a telephone interview every 3 months. The total follow-up duration was 12 months after first admission.

### Statistical Analysis

Data were presented as mean ± standard deviation (SD) if the continuous variables were distributed normally according to the Kolmogorov–Smirnov test. Otherwise, data were presented as median [interquartile range (IQR)]. Classification variables were expressed as frequency (percentage, %). The differences between the two groups were analyzed using the Student's *t*-test and Wilcoxon test for normally and abnormally distributed data, respectively. Categorical data were compared using the chi-square test when comparing numbers ≥5 or Fisher's exact test when comparing small numbers <5. Univariate logistic regression analysis was used to screen factors that might affect highly active NMOSD outcomes. Multivariate logistic regression was used to analyze the independent effects of variables on highly active relapse outcomes. Variables with *P* < 0.2 according to univariate logistic regression analysis or variables considered to be associated with relapse outcomes in NMOSD in previous studies, such as age, sex, hypertension, and initial EDSS, were included in the multivariate model. Receiver operating characteristic (ROC) curve analysis was performed to test the predictive ability of Hcy and the 24h IgG synthesis rate for highly active NMOSD and predict the optimal cut-off value of Hcy and the 24h IgG synthesis rate in patients with highly active NMOSD. *P* < 0.05 was considered significant. All analyses were performed using IBM SPSS Statistics 21 software and the diagram was generated with GraphPad Prism 8.

## Results

### Demographic and Clinical Characteristics

**Figure 2:**
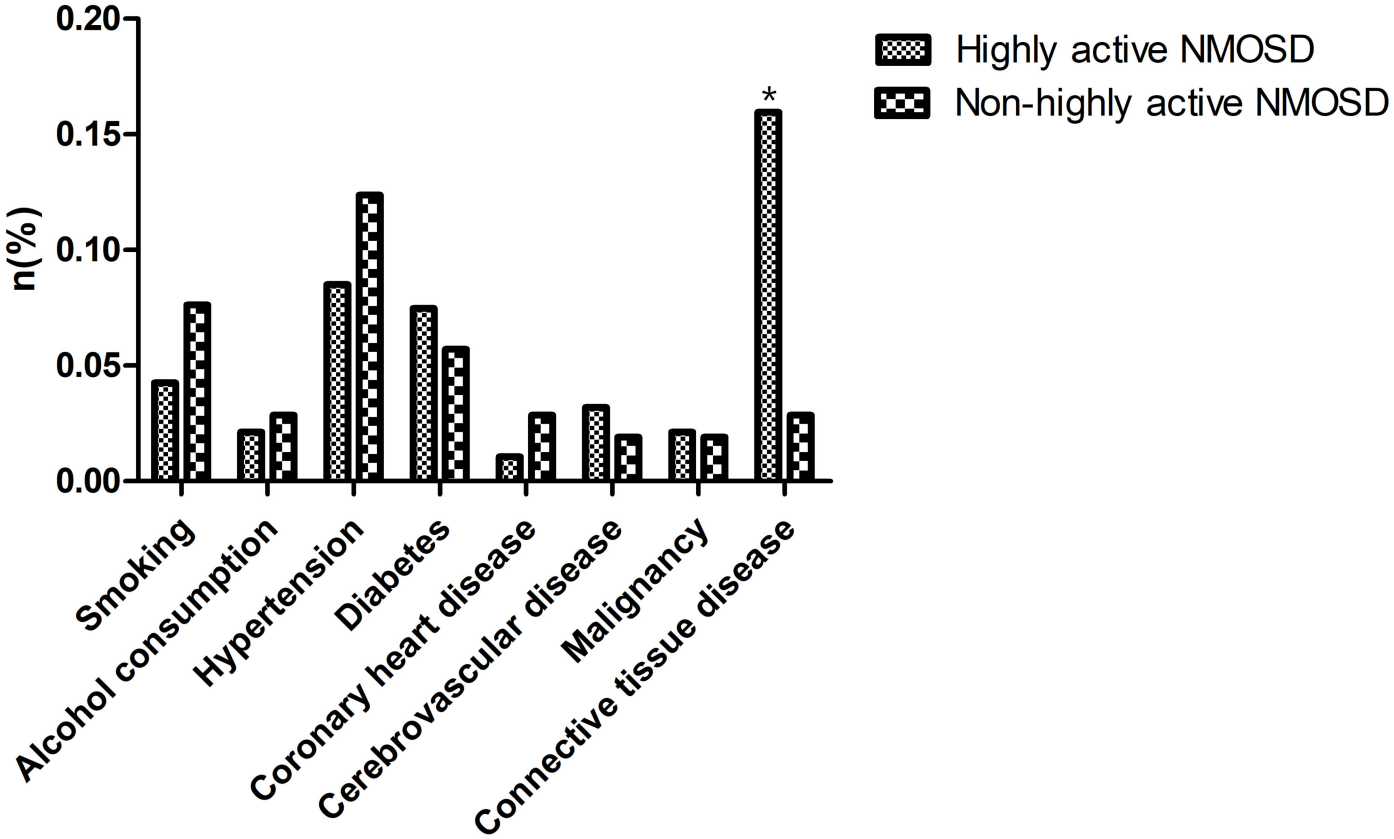
Comorbidities of the patients. The prevalence of connective tissue disease was significantly higher among the patients with highly active NMOSD than non-highly active NMOSD. *P < 0.05.

In the database of the First Affiliated Hospital of Zhengzhou University from January 2013 to December 2019, 1,351 patients met the NMOSD diagnostic criteria. Of the 199 cases of AQP4-IgG positive first-attack NMOSD who were admitted to our hospital, 94 met the inclusion criteria for highly active NMOSD in accordance with the specified relapse times during the 12 month-follow-up after the first attack, and 105 AQP4-IgG positive cases were enrolled as controls (non-highly active NMOSD). Patients with highly active NMOSD experienced an average of 2.3 relapses during the 12-month follow-up period. As shown in Table 1, there were no significant differences in age, gender, and the number of patients who smoke and consume alcohol between the two groups (*P* > 0.05). The prevalence of hypertension, diabetes, coronary heart disease, cerebrovascular disease, and malignancy was not significantly different between the two groups (*P* > 0.05). The prevalence of connective tissue disease was significantly higher among the patients with highly active NMOSD than non-highly active NMOSD (15.96% for patients with highly active NMOSD, 2.86% for controls, *P* = 0.002). In highly acive NMOSD group, 15 cases presented with connective tissue disease (three cases with systemic lupus erythematosus, three cases with rheumatoid arthritis, and nine cases with Sjögren syndrome). In the non-highly acive NMOSD group, three cases presented with connective tissue disease (one cases with systemic lupus erythematosus, two cases with Sjögren syndrome) (Figure 2).

**Table 1:** Comparison of clinical data between patients with highly active NMOSD and non-highly active NMOSD patients.

	**Total patients** ** (*n* = 199)**	**Highly active** ** NMOSD (*n* = 94)**	**Non-highly active** ** NMOSD (*n* = 105)**	***P-*value**
Age in years (mean ± SD)	41.02 ± 15.49	39.88 ± 16.85	42.04 ± 14.17	0.333
Gender, female, *n* (%)	171 (85.93)	81 (86.17)	90 (85.71)	0.926
Smoking, *n* (%)	12 (6.03)	4 (4.26)	8 (7.62)	0.382
Alcohol consumption, *n* (%)	5 (2.51)	2 (2.13)	3 (2.86)	1.000
**Comorbidities**				
Hypertension, *n* (%)	21 (10.55)	8 (8.51)	13 (12.38)	0.375
Diabetes, *n* (%)	13 (6.53)	7 (7.47)	6 (5.71)	0.621
Coronary heart disease, *n* (%)	4 (2.01)	1 (1.06)	3 (2.86)	0.624
Cerebrovascular disease, *n* (%)	5 (2.51)	3 (3.19)	2 (1.90)	0.669
Malignancy, *n* (%)	4 (2.01)	2 (2.13)	2 (1.90)	1.000
^a^Connective tissue disease, *n* (%)	17 (8.54)	15 (15.96)	3 (2.86)	0.002^*^
**Onset attack**, ***n*****(%)**				
Optic neuritis	72 (36.18)	40 (42.55)	32 (30.48)	0.077
Transverse myelitis	148 (74.37)	70 (74.47)	78 (74.29)	0.977
Initial EDSS	5.16 ± 1.84	5.33 ± 1.75	5.02 ± 1.91	0.235
**Treatment**, ***n*****(%)**				
Corticosteroid	186 (93.47)	88 (93.62)	98 (93.33)	0.936
Intravenous immunoglobulin	16 (8.04)	9 (9.57)	7 (6.67)	0.463
Immunosuppressant	66 (33.17)	31 (32.98)	35 (33.33)	0.92
Azathioprine	41 (20.60)	19 (20.21)	22 (20.95)	0.898
Mycophenolate mofetil	21 (10.55)	10 (10.64)	11 (10.48)	0.97
Rituximab	2 (1.01)	1 (1.06)	1 (0.95)	1.000
Methotrexate	2 (1.01)	1 (1.06)	1 (0.95)	1.000
Final EDSS	4.633 ± 1.93	5.42 ± 1.66	3.92 ± 1.89	<0.001^*^

a *Connective tissue disease consists of systemic lupus erythematosus, rheumatoid arthritis, and Sjögren syndrome*.

* *P < 0.05*.

Clinical symptoms of all patients were evaluated at the time of their first attack before treatment and recorded as an initial EDSS score, which did not differ significantly between the groups. Patients received different treatments according to their clinical symptoms and financial situation, such as corticosteroids, immunoglobulin, and immunosuppressants (azathioprine, mycophenolate mofetil, methotrexate, and rituximab). There were no statistically significant differences in these parameters between the two groups. At the final follow-up, the final EDSS score of the highly active NMOSD group was significantly higher than that of the controls (*P* < 0.001; Table 1).

Regarding the laboratory test results of the study groups (Table 2), there were no significant differences in the erythrocyte, hemoglobin, lymphocyte, and glycosylated hemoglobin, folic acid, and vitamin B12 of the two groups. The proportion of patients with hepatic dysfunction, renal dysfunction, high triglyceride and total cholesterol levels, and positive thyroid peroxidase or thyroglobulin antibodies was similar between the two groups. The proportion of ANA-positive patients was higher in the highly active NMOSD group than in the control group. Leukocyte counts, Hcy levels, CSF leukocyte counts and protein concentration, IgG index, and 24h IgG synthesis rate were higher in the highly active NMOSD group than in the control group.

**Table 2:** Comparison of laboratory tests between patients with highly active NMOSD and non-highly active NMOSD patients.

	**Total patients** ** (*n* = 199)**	**Highly active NMOSD** ** (*n* = 94)**	**Non-highly active NMOSD** ** (*n* = 105)**	***P*-value**
Leukocyte counts, median (IQR) (×10^9^/L)	6.4 (5.0–7.9)	6.7 (5.5–8.9)	6.0 (4.7–7.5)	0.036^*^
Erythrocyte counts, median (IQR) (×10^12^/L)	4.18 (3.91–4.52)	4.15 (3.93–4.97)	4.18 (3.90–4.56)	0.319
Hemoglobin, median (IQR) (g/L)	125 (116–135)	126 (116–134.5)	125 (116–135)	0.787
Lymphocyte counts, median (IQR) (×10^9^/L)	1.79 (1.23–2.48)	1.82 (1.315–2.67)	1.72 (1.16–2.35)	0.385
Glycosylated hemoglobin, median (IQR)	5.70 (5.40–6.08)	5.77 (5.36–6.13)	5.67 (5.4–5.91)	0.209
^a^Hepatic dysfunction, *n* (%)	17 (8.54)	10 (10.64)	7 (6.67)	0.317
^b^ Renal dysfunction, *n* (%)	4 (2.01)	2 (2.13)	2 (1.90)	1.000
High triglyceride, *n* (%)	31 (15.58)	13 (13.83)	18 (17.14)	0.520
High total cholesterol, *n* (%)	41 (20.60)	17 (18.09)	24 (22.86)	0.406
Homocysteine levels, median (IQR) (μmol/L)	11.95 (9.27–16.26)	14.03 (9.89–18.83)	10.87 (8.94–13.32)	<0.001^*^
Folic acid, median (IQR) (ng/mL)	7.3 (4.70–11.23)	6.5 (4.48–10.45)	7.78 (5.08–11.45)	0.208
Vitamin B12, median (IQR) (pg/mL)	516.2 (346.0–788.7)	555.65 (337.25–836.85)	467 (356–728.2)	0.222
ANA-positive, *n* (%)	39 (19.60)	25 (26.60)	14 (13.33)	0.019^*^
Thyroid peroxidase / thyroglobulin antibodies positive, *n* (%)	17 (8.84)	8 (8.51)	9 (8.57)	0.988
Cerebrospinal fluid leukocyte counts, median (IQR) (×10^6^/L)	6 (2–19.25)	12 (6–25)	4 (2–11)	<0.001^*^
Cerebrospinal fluid protein concentration, median (IQR) (mg/L)	396.4 (284–582.6)	460.85 (328.45–653.25)	346.1 (235.55–503.5)	<0.001^*^
IgG index, median (IQR)	0.63 (0.53–0.78)	0.715 (0.58–0.84)	0.58 (0.51–0.71)	<0.001^*^
24h IgG synthesis, median (IQR) (mg/24h)	3.12 (0–10.23)	7.74 (2.97–15.22)	1.37 (0–3.87)	<0.001^*^

a *Hepatic dysfunction refers to abnormal transaminase and bilirubin level*.

b *Renal dysfunction refers to abnormal creatinine and urea nitrogen level*.

* *P < 0.05*.

### Predictors Associated With the Occurrence of Highly Active NMOSD

To explore potential risk factors that may predict the occurrence of highly active NMOSD, univariate logistic regression analysis was performed. Analyses showed that connective tissue disease [OR (odds ratios) = 6.456, 95% CI (Confidence interval): 1.806–23.078, *P* = 0.004], serum leukocyte counts (OR = 1.119, 95% CI: 1.012–1.238, *P* = 0.028), serum Hcy levels (OR = 1.095, 95% CI: 1.039–1.154, *P* = 0.001), ANAs (OR = 2.355, 95% CI: 1.14–4.864, *P* = 0.021), CSF protein concentration (OR = 1.001, 95% CI: 1.000–1.003, *P* = 0.016), 24h IgG synthesis rate (OR = 1.058, 95%CI: 1.022–1.096, *P* = 0.002) were significantly correlated to the occurrence of highly active NMOSD (Table 3).

**Table 3:** Univariate logistic regression analysis of potential risk factors that may predict high relapse events in patients with NMOSD.

**Variables**	**Univariate analysis**	
	**OR (95% CI)**	***P*-value**
Age	0.991 (0.973–1.009)	0.327
Gender, female	1.038 (0.466–2.314)	0.926
Smoking	0.539 (0.157–1.851)	0.326
Drinking	0.739 (0.121–4.522)	0.744
Hypertension	0.658 (0.260–1.666)	0.378
Diabetes	1.328 (0.430–4.101)	0.622
Coronary heart disease	0.366 (0.037–3.576)	0.387
Cerebrovascular disease	0.589 (0.096–3.604)	0.567
Malignancy	1.12 (0.155–8.109)	0.911
^a^Connective tissue disease	6.456 (1.806–23.078)	0.004^*^
Optic neuritis	1.69 (0.943–3.027)	0.078
Transverse myelitis	1.01 (0.534–1.91)	0.977
Initial EDSS	1.097 (0.942–1.278)	0.234
Corticosteroid	1.048 (0.339–3.236)	0.936
Intravenous immunoglobulin	1.467 (0.524–4.109)	0.466
Immunosuppressant	0.97 (0.537–1.753)	0.920
Leukocyte counts	1.119 (1.012–1.238)	0.028^*^
Erythrocyte counts	0.635 (0.374–1.077)	0.092
Hemoglobin	0.002 (0.974–1.009)	0.347
Lymphocyte counts	1.123 (0.826–1.528)	0.460
Glycosylated hemoglobin	1.161 (0.782–1.722)	0.460
Hepatic dysfunction	1.667 (0.608–4.571)	0.321
Renal dysfunction	1.12 (0.155–8.109)	0.911
High triglyceride	0.776 (0.357–1.684)	0.521
High total cholesterol	0.745 (0.372–1.493)	0.407
Homocysteine levels	1.095 (1.039–1.154)	0.001^*^
Folic acid	0.977 (0.917–1.040)	0.464
Vitamin B12	1.000 (1.000–1.001)	0.240
ANAs positive	2.355 (1.14–4.864)	0.021^*^
Thyroid peroxidase/thyroglobulin antibodies positive	0.992 (0.367–2.686)	0.988
Cerebrospinal fluid leukocyte counts	1.006 (0.997–1.015)	0.187
Cerebrospinal fluid protein concentration	1.001 (1.000–1.003)	0.016^*^
IgG index	1.555 (0.82–2.948)	0.176
24h IgG synthesis rate	1.058 (1.022–1.096)	0.002^*^

a *Connective tissue disease consists of systemic lupus erythematosus, rheumatoid arthritis, and Sjögren syndrome*.

* *P < 0.05*.

**Figure 3:**
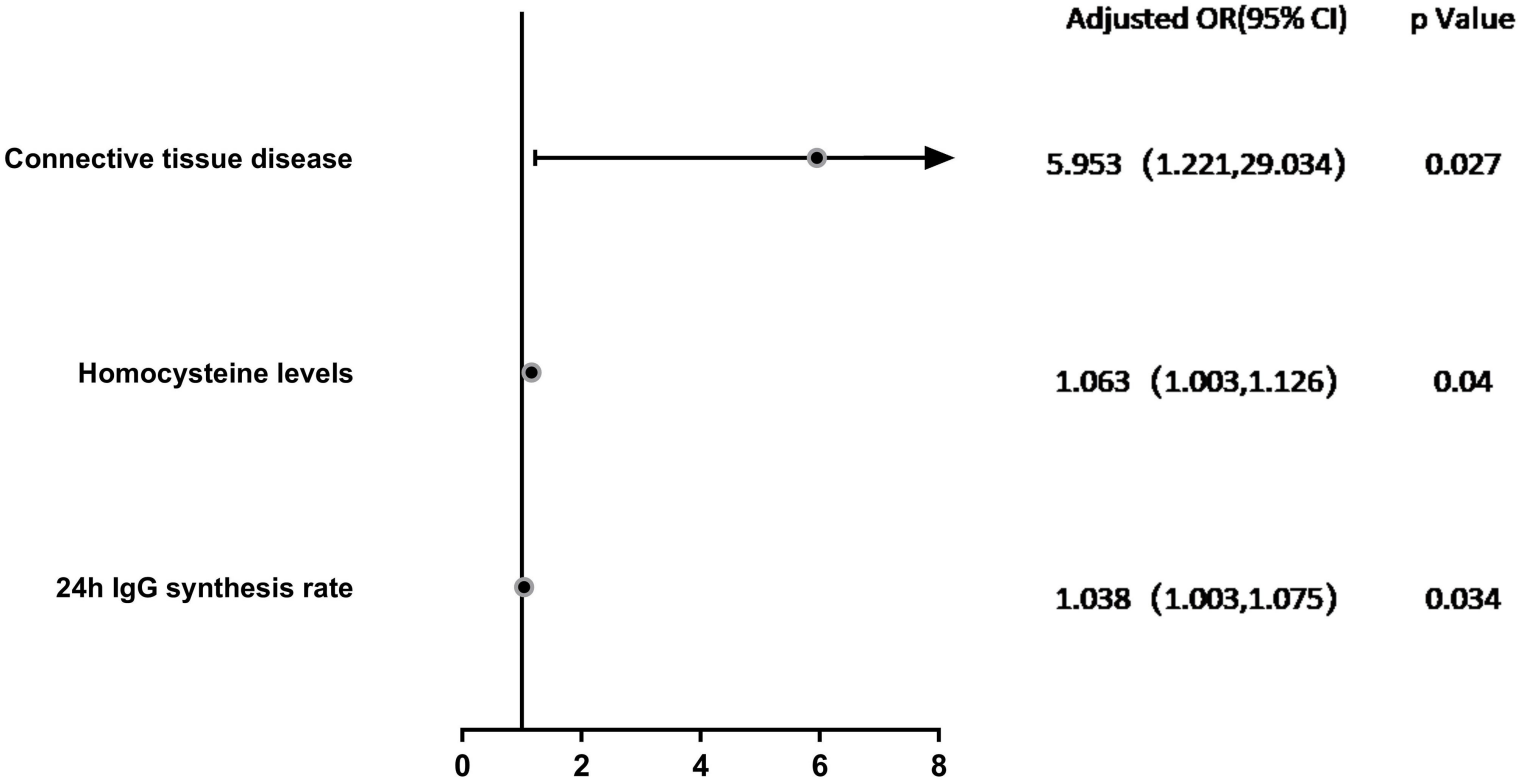
Forest Plot. Multivariate logistic regression analysis of factors associated with highly active NMOSD in Adjust I model. Connective tissue disease (OR = 5.953, 95% CI: 1.221–29.034, *P* = 0.027), Hcy levels (OR = 1.063, 95% CI: 1.003–1.126, *P* = 0.04), and 24h IgG synthesis rate (OR = 1.038, 95% CI: 1.003–1.075, *P* = 0.034) were significantly correlated with the occurrence of highly active NMOSD.

Variables with a significance of *P* < 0.2 according to univariate logistic regression analysis or variables considered to be associated with relapses in NMOSD in previous studies, such as age, gender, hypertension, and initial EDSS, were included in the multivariate model. Multivariate logistic regression analysis showed that connective tissue disease (OR = 5.953, 95% CI: 1.221–29.034, *P* = 0.027), Hcy levels (OR = 1.063, 95% CI: 1.003–1.126, *P* = 0.04), and 24h IgG synthesis rate (OR = 1.038, 95% CI: 1.003–1.075, *P* = 0.034) were significantly correlated with the occurrence of highly active NMOSD (Table 4; Figure 3).

**Table 4:** Multivariate logistic regression analysis of potential risk factors to predict high relapse events in patients with NMOSD.

	**Multivariate analysis**			
**Variables**	^**a**^ **Basic model**		^**b**^ **Adjust I model**	
	**OR (95% CI)**	***P*-value**	**OR (95% CI)**	***P-*value**
Age			0.993 (0.971–1.017)	0.577
Gender, female			0.905 (0.39–2.416)	0.841
Hypertension			0.578 (0.18–1.856)	0.357
^c^Connective tissue disease	5.009 (1.095–22.915)	0.038^*^	5.953 (1.221–29.034)	0.027^*^
Optic neuritis			1.708 (0.875–3.336)	0.117
Initial EDSS			1.003 (0.825–1.219)	0.979
Leukocyte counts	1.086 (0.971–1.214)	0.147	1.12 (0.991–1.267)	0.07
Erythrocyte counts			0.582 (0.294–1.152)	0.12
Homocysteine levels	1.077 (1.020–1.137)	0.008^*^	1.063 (1.003–1.126)	0.04^*^
ANAs positive	1.149 (0.44–3.003)	0.777	0.853 (0.298–2.439)	0.766
Cerebrospinal fluid leukocyte counts			1.005 (0.995–1.015)	0.349
Cerebrospinal fluid protein concentration	1.001 (0.999–1.002)	0.468	1.001 (0.999–1.002)	0.419
IgG index			1.24 (0.651–2.362)	0.514
24h IgG synthesis rate	1.045 (1.010–1.082)	0.012^*^	1.038 (1.003–1.075)	0.034^*^

a *Basic Model: Variables with P <0.05 in the univariate logistic regression analysis were included in the multivariate model*.

b *Adjust I model: Variables with P < 0.2 in the univariate logistic regression analysis or variables considered to be associated with NMOSD relapse outcomes in previous studies, such as age, gender, hypertension, and initial EDSS, were included in the multivariate model*.

c *Connective tissue disease consists of systemic lupus erythematosus, rheumatoid arthritis, and Sjögren syndrome*.

* *P < 0.05*.

### ROC Curve for Hcy and 24h IgG Synthesis Rate at Admission Predicts the Occurrence of Highly Active NMOSD

**Figure 4:**
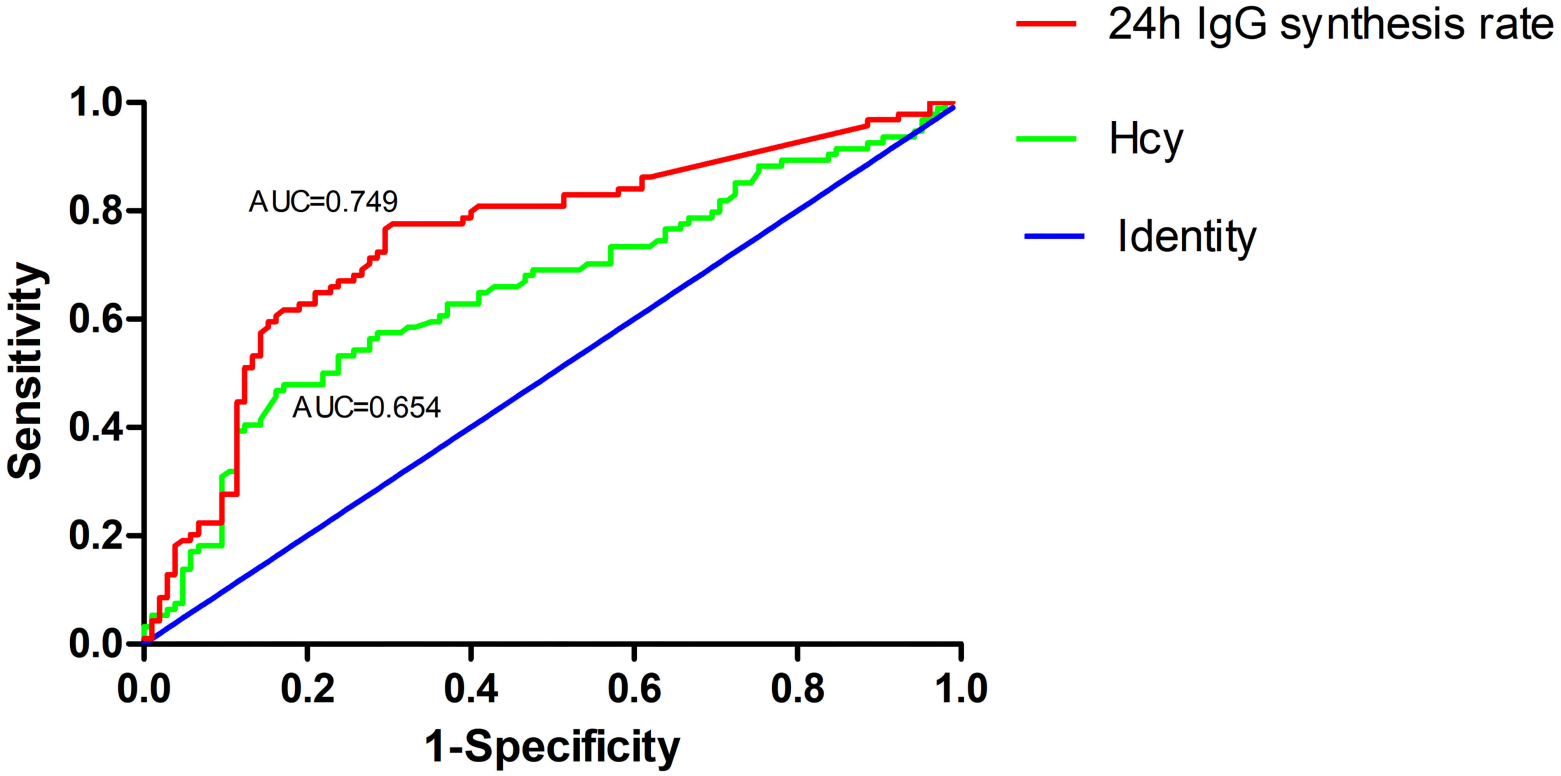
Receiver operating characteristic (ROC) curve showing the predictive ability of Hcy and 24h IgG synthesis rate for highly active NMOSD. The area under the ROC curve was 0.654 and 0.749 for Hcy levels and 24h IgG synthesis rates, respectively.

ROC curve analysis was used to evaluate the predictive value of Hcy and 24h IgG synthesis rate in patients with highly active NMOSD. The area under the ROC curve was 0.654 (95% CI: 0.577–0.731, *P* < 0.001) and 0.749 (95% CI: 0.679–0.819, *P* < 0.001) for Hcy levels and 24h IgG synthesis rates, respectively. At an Hcy cut-off value of 14.735 μmol/L, the sensitivity for predicting the occurrence of highly active NMOSD was 47.9%, and the specificity was 82.9%. At a 24h IgG synthesis rate cut-off value of 2.815, the sensitivity for predicting highly active NMOSD was 77.7%, and the specificity was 69.5% (Figure 4).

## Discussion

NMOSDs are severe inflammatory disorders of the central nervous system that mainly affect the optic nerves and spinal cord, which causes severe and irreversible disabilities (14, 15), and limited reliable prognostic indicators currently exist. In this study, we performed a retrospective analysis to explore independent risk factors among patients with highly active NMOSD compared to those with non-highly active NMOSD. We analyzed patients' clinical characteristics, laboratory test results, and relapse information and found that the prevalence of connective tissue disease, leukocyte counts, Hcy levels, the proportion of ANA-positive patients, CSF leukocyte counts, protein concentration, IgG index, and 24h IgG synthesis rate were higher in the highly active NMOSD group than among the non-highly active NMOSD patients. Logistics regression analysis indicated that the prevalence of connective tissue disease, Hcy levels, and 24h IgG synthesis rate might be independent risk factors for highly frequent relapse events in patients with first-attack NMOSD during the 12 month follow-up period. The optimal cut-off values of Hcy and the 24h IgG synthesis rate for predicting highly active NMOSD were 14.735 μmol/L and 2.815 mg/24h, respectively. To the best of our knowledge, this is the first study to investigate the relationship between the prevalence of comorbidities, laboratory results, and relapse events in NMOSD and to determine whether these indicators are risk factors among patients with highly active NMOSD in a real-world setting. In order to eliminate the effects of previous treatments (such as glucocorticoids, immunoglobulin, and immunosuppressants) and accurately calculate the times of relapse events during the follow-up period, we focused on patients with first-attack NMOSD in the study.

In the database of the First Affiliated Hospital of Zhengzhou University from January 2013 to December 2019, 1,351 patients were preliminarily enrolled, and 1,290 patients met the NMOSD diagnostic criteria, therefore, we collected their data. After our strict screening process, 199 cases of first-attack NMOSD were included in the study. Prior evidence suggests that the female-to-male ratio among NMOSD patients is about 8:1 for AQP4-IgG seropositive patients and 2:1 for AQP4-IgG seronegative patients (16). Our findings showed that, among cases of highly active NOMSD, the proportion of females was higher (6.23:1) compared to that of the previous report (3.95:1) (4). However, the logistic regression analysis results indicated that sex did not significantly affect highly active NMOSD (OR = 1.095, 95% CI: 0.403–2.974, *P* = 0.859). We speculate that different survey regions may account for the difference in the female-to-male ratio. In this study, we mainly focused on the population of the central region of China, taking the geographical location of our hospital into account. The mean age of patients with highly active NMOSD was 39.88, similar to that of previous studies.

Previous studies have demonstrated that comorbidities such as vascular disease, may potentially explain the heterogeneity in multiple sclerosis (MS) outcomes (17). It was also reported that the comorbidity burden was greater among patients with highly active NMOSD than among the overall NMOSD population (4). Thus, we investigated whether comorbidities are associated with relapse and progression in NMOSD. Previous studies have shown that the most common comorbidities that coexist with NMOSD are autoimmune diseases, such as systemic lupus erythematosus, Sjögren syndrome, rheumatoid arthritis, and others (18–21). Our results were consistent with those of previous reports in that we found that the prevalence of connective tissue diseases was greater among patients with highly active NMOSD than in patients with non-highly active NMOSD. Furthermore, the multivariate analysis results showed that connective tissue diseases were independent risk factors for highly active NMOSD, suggesting that connective tissue diseases may be associated with a high risk of relapse in NMOSD. One possible mechanism is the activation of autoreactive Th1 cells and B cells via Th2 cells in autoimmune diseases (22). On the other hand, it is believed that ANAs are the most common biomarkers of these connective tissue diseases, which could cause inflammation and tissue damage by cross-reactivity and forming immune complexes of antibodies with DNA or nucleosomes. The proportion of ANA-positive patients is between 27.3 and 82.6% (23–25), whereas in our study, the proportion was 19.6%, which is lower than that of previous reports. Since ANA tests were not included in the conventional examination, many patients without ANA results were excluded during the screening process, which may account for the lower proportion of ANA-positive patients in our study. ANAs are reportedly related to more severe disease activity in patients with NMOSD according to a previous study (7). In our study, univariate analysis also revealed that ANAs were risk factors in patients with highly active NMOSD (OR = 2.355, 95% CI: 1.1–4.864, *P* = 0.021), but no significant correlation was found during multivariate analysis.

The role of serum Hcy in autoimmune demyelinating diseases of the central nervous system has recently attracted more attention. An updated meta-analysis indicated that elevated Hcy levels might affect the pathogenesis or progression of MS. The prognosis of MS patients with hyperhomocysteinemia was worse than that of MS patients with lower Hcy levels in terms of disease progression (26). Hcy levels are associated with the progression of NMOSD. A previous study reported that serum Hcy levels were higher in patients with acute-phase NMOSD who had severe initial symptoms (EDSS score ≥ 4) than in patients with mild initial symptoms (EDSS score < 4), and EDSS was positively correlated with Hcy levels in acute-phase NMOSD (7). In our study, we found that serum Hcy levels were higher in the patients with highly active NMOSD than in patients with non-highly active NMOSD. Moreover, Hcy level was an independent risk factor among patients with highly active NMOSD, suggesting that those with higher Hcy levels may have a higher relapse rate during the first 12 months following the first attack. This is the first study to investigate Hcy levels in patients with highly active NMOSD and the relationship between Hcy levels and relapse events.

The effect of Hcy in highly active NMOSD is still not clear. Possible underlying mechanisms of higher Hcy leading to relapse are presented as follows. Elevated Hcy levels can cause oxidative stress, and mitochondrial dysfunction by increasing ROS production (27, 28). Hcy is an agonist of glutamate receptors [N-methyl-D-aspartate receptor (NMDAR)], the stimulation of which can cause excitotoxicity, the activation of caspases and increase of the intracellular calcium concentration, inducing neuronal injury and apoptosis. Hcy can exert pro-inflammatory effects by modulation of adaptive immune system cell function and disturbing the blood brain barrier (BBB). Th17-cells have been identified to be able to destroy BBB by producing pro-inflammatory IL-17 and migrate into CNS by expressing chemokine receptor CCR-6 (CD196). Hcy can affect the BBB functioning through activating the Th17-immune response. In addition (29). Hcy plays an important role in structural instability and degeneration of myelin sheath by inhibiting methyl donors, which may have an adverse effect on disease progression (30–32).

A double-blinded clinical trial demonstrated that administering vitamin B12 supplements and folate might reduce serum Hcy levels in MS patients and improve the physical and mental aspects of their quality of life (33, 34). Based on our results, we recommend that more positive treatment strategies should be considered for patients with NMOSD who have higher Hcy levels to reduce the occurrence of relapse events.

The normal range of intrathecal IgG synthesis is between −9 and 3.3 mg/24h (35). An elevated IgG synthesis rate has been reported in over 90% of patients with clinically definite MS. Considerable evidence indicates that IgG synthesis reflects the degree of chronic inflammation in the central nervous system and can be used as a monitoring marker for the progression of MS (35). Quantification of CSF oligoclonal bands (OBs) is a prognostic indicator in MS; patients with reduced or no OBs tend to have a better prognosis (36). Since the IgG daily synthesis rate was strongly correlated to OBs, we examined whether IgG synthesis predicts the prognosis of NMOSD. We found that the intrathecal IgG synthesis rate was higher in patients with highly active NMOSD than in patients with non-highly active NMOSD. We further demonstrated that a higher IgG synthesis rate may be related to a higher possibility of relapse in NMOSD.

This study has several limitations that should be noted. First, the total number of subjects included in the analyses was small, and the patients were from a single center. Also, we mainly focused on patients with highly active NMOSD who experienced two or more clinical relapses within 12 months following the first attack, and the follow-up period was relatively short. The results need to be further validated in larger, multicenter studies with longer follow-up periods. Second, quantitative analysis of OBs is not performed at our hospital, therefore, we failed to include this parameter in our study, which may be a source of bias in our results. Finally, we only included AQP4-IgG positive patients in the study, and the risk factors should also be studied in AQP4-IgG negative patients to analyze the results more accurately.

In conclusion, we demonstrated that connective tissue disease comorbidity, Hcy levels, and 24h IgG synthesis rate may be independent risk factors in patients with AQP4-IgG positive highly active NMOSD. High Hcy levels and 24h IgG synthesis rates are associated with a high relapse rate in these patients after the first attack, which suggests that more positive treatment should be applied when these abnormal indicators are encountered to reduce relapse events in patients with NMOSD. Further studies with more data are needed to validate these conclusions.

## Data Availability Statement

The original contributions presented in the study are included in the article/Supplementary Material, further inquiries can be directed to the corresponding author/s.

## Ethics Statement

The studies involving human participants were reviewed and approved by Ethics Committee of the First Affiliated Hospital of Zhengzhou University (2019-KY-018). The patients/participants provided their written informed consent to participate in this study.

## Author Contributions

YL: methodology, formal analysis, data curation, writing — original draft, writing-review and editing. JZ: investigation, writing — review and editing. YZ: methodology, investigation, writing—review and editing. HX: investigation, writing — review and editing. RD and YY: formal analysis. LJ and JT: data curation. YJ: conceptualization, methodology, supervision, and funding acquisition. The first draft of the manuscript was written by YL. All authors commented on previous versions of the manuscript and read and approved the final manuscript.

## Conflict of Interest

The authors declare that the research was conducted in the absence of any commercial or financial relationships that could be construed as a potential conflict of interest.

## Publisher's Note

All claims expressed in this article are solely those of the authors and do not necessarily represent those of their affiliated organizations, or those of the publisher, the editors and the reviewers. Any product that may be evaluated in this article, or claim that may be made by its manufacturer, is not guaranteed or endorsed by the publisher.

## Abbreviations

NMOSD: neuromyelitis optica spectrum disorder
AQP-4: Aquaporin-4
ANA: Antinuclear antibody
EDSS: Expanded Disability Status Scale
Hcy: Homocysteine
CI: Confidence interval
OR: Odds ratios
CSF: Cerebrospinal fluid
SD: Standard deviation
IQR: Interquartile range
OB: Oligoclonal band
NMDAR: N-methyl-D-aspartate receptor
BBB: Blood brain barrier.
